# The Human Factors and Ergonomics of P300-Based Brain-Computer Interfaces

**DOI:** 10.3390/brainsci5030318

**Published:** 2015-08-10

**Authors:** J. Clark Powers, Kateryna Bieliaieva, Shuohao Wu, Chang S. Nam

**Affiliations:** 1 Department of English, North Carolina State University, Raleigh, NC 27695, USA; E-Mails: jcpower2@ncsu.edu (J.C.P.); kbielia@ncsu.edu (K.B.); 2 Fitts Department of Industrial and Systems Engineering, North Carolina State University, Raleigh, NC 27695, USA; E-Mail: swu6@ncsu.edu

**Keywords:** brain-computer interface, P300, human factors and ergonomics, oddball, assistive technology, user acceptance

## Abstract

Individuals with severe neuromuscular impairments face many challenges in communication and manipulation of the environment. Brain-computer interfaces (BCIs) show promise in presenting real-world applications that can provide such individuals with the means to interact with the world using only brain waves. Although there has been a growing body of research in recent years, much relates only to technology, and not to technology in use—*i.e.*, real-world assistive technology employed by users. This review examined the literature to highlight studies that implicate the human factors and ergonomics (HFE) of P300-based BCIs. We assessed 21 studies on three topics to speak directly to improving the HFE of these systems: (1) alternative signal evocation methods within the oddball paradigm; (2) environmental interventions to improve user performance and satisfaction within the constraints of current BCI systems; and (3) measures and methods of measuring user acceptance. We found that HFE is central to the performance of P300-based BCI systems, although researchers do not often make explicit this connection. Incorporation of measures of user acceptance and rigorous usability evaluations, increased engagement of disabled users as test participants, and greater realism in testing will help progress the advancement of P300-based BCI systems in assistive applications.

## 1. Introduction

Brain-computer interfaces (BCIs) have been investigated for decades as a means to enable users with profound neuromuscular impairment [1]. From initial steps, such as those by Vidal (1973) [2] and Elbert *et al.* (1980) [3] using electroencephalography (EEG), research into BCIs and their applications has grown rapidly in the decades since, with hundreds of peer-reviewed studies now being published annually [4]. These studies use a variety of terminology, including brain-machine interface, brain interface, neural prosthetic, neural interface, and so forth, but they are all united in that the various approaches do not rely on peripheral nerves or muscles, but rather on signals from the central nervous system, to enable communication or control [5].

Beyond the admittedly tantalizing possibility of being able to interact with the world using the mind alone (which is quite an overstatement, as the user and the BCI must be trained to work in concert [4]), BCI technology holds great potential in that it is perhaps the only possibility for enabling individuals without voluntary motor function, but with cognitive function, to interact with their external environment [5,6,7,8,9]. BCI has been proposed as an assistive technology (and we consider therapeutic applications to be implicitly assistive) for individuals with disabilities such as those resulting from autism [10,11,12], aphasia [13,14], brainstem stroke [15,16], spinal cord injury [17,18,19], and neurodegenerative diseases [20,21,22], among other afflictions and challenges [23]. However, research has put special attention on individuals with amyotrophic lateral sclerosis (ALS) [24], as this condition impacts voluntary muscle control while leaving cognitive function intact, making such patients an obvious group to benefit from potential BCI-based assistive technologies. That said, this clinical group is relatively small, with ALS having a prevalence of about six per 100,000 people [25], although geographic distribution is not uniform. A 2010–2011 survey by the National ALS Registry recorded 12,187 persons in the United States living with ALS [26].

Among the systems that have been developed to implement BCI, one category is based upon sensing the P300 wave, which is an event-related potential component associated with unexpected stimuli that provide task-relevant information [27,28]. Unexpected stimuli that are not task-relevant produce a similar component, but that component can be discriminated by a slightly lower latency [29]. The P300 component can thus be associated with information processing [30], decision-making [31], and intentionality [32]. Furthermore, the P300 wave has been the focus of much BCI research because of its ease of evocation and consistency [29]. Furthermore, the evocation of the P300 wave requires relatively little initial training of the subject [33]. 

The P300 takes its name from its polarity and latency—approximately 300 ms after a triggering event, the P300 wave presents a positive voltage peak [34]. As neural stimulation produces electrical activity that can be sensed by non-invasive means, P300-based BCIs capitalize on discriminating this voltage peak from background signal noise, and associating the desired signal with an event. The most common evocation method of the P300 wave is known as the “oddball” paradigm. The method involves a discrimination task. Two stimuli are presented, one being the non-target stimulus, and the other being the target stimulus. The name “oddball” arises because the latter stimulus is infrequent relative to the former stimulus. The subject is to discriminate between the two, that is to respond to the target stimulus but not to the non-target stimulus. The stimulus can take many forms, but is commonly visual or auditory [35]. The acquisition of the resulting potential from the target stimulus, and the association of that stimulus with an event, are what allow for subject control of a P300-based BCI using the oddball paradigm. The P300-based BCI, although first described more than 25 years ago [36], has only received significant attention in recent years. It is now one of the prominent types of BCI being developed [33].

## 2. Review Objectives

This review is concerned with the human factors and ergonomics (HFE) of P300-based BCIs. By HFE we wish to indicate the concern with the cognitive and physical characteristics of human-machine systems, and attending to them through study and design, so as to optimize human well-being and system performance [37]. In relation to BCIs, we are concerned with HFE issues such as interface design, usability, accessibility (across clinical and non-clinical groups), cognitive load, affective response, and so forth [6,37,38]. P300-based BCIs, and BCIs generally, have received insufficient attention in terms of HFE. This is largely due to the fact that the technology is still relatively new, and the bulk of research is directed at the technological underpinnings of the systems [39]. However, we cannot neglect the needs of users, and specifically of different user types [16]. For example, Nam *et al.* 2012 [40] found significant differences in the BCI performance of healthy *vs.* disabled subjects, and furthermore differences among disabled subjects depending on severity of impairment. Even more importantly, HFE play a critical role not only in how a subject interacts with a BCI itself, but rather how a BCI can be incorporated successfully into a subject’s preexisting physical and social context. For example, Blain-Moraes *et al.* (2012) [41] conducted a focus group with ALS patients and their caregivers who jointly had experience with BCI use. The participants reported that while personal factors, such as physical, physiological, and psychological factors, were important for them in accepting the use of BCI-based assistive technologies, of greater importance were relational factors, such as corporeal (the subjects’ bodily relation with the equipment, such as electrodes and screens), technological (the subject’s relationship and attitude towards technology generally) and social (crucially, how BCI technologies and their human support networks integrate with users and their human support networks). Together, the micro-level perspective (BCI systems) and the macro-level perspective (BCI systems integrating into established caregiving and life-leading systems) underline the essential consideration of HFE in the development of, research on, and eventual deployment of BCI-based assistive technologies [41].

As such, our primary concern was P300-based BCI as an assistive technology, that is to say a technology that provides ability to users with disability. As such, HFE are an implicit concern. To make this concern explicit, we collected and assessed research relevant to HFE of P300-based BCIs. Of interest were system characteristics or implementations that could, among other things, improve user performance (in reducing error or increasing speed), reduce training needs, reduce fatigue and stress, and increase overall ease of use. Uniting these interests was the question of user acceptance, and how best to measure such acceptance. As HFE relevance to P300-based BCI is an overly broad metric, we focused on three research questions to guide our review. These questions do not exhaust the HFE relevance to P300-based BCI, nor do they cover the entire spectrum of relevant HFE concerns. Rather, as this was an exploratory review of a relatively small subset of the literature, these questions were designed to “sample” the spectrum of relevant HFE concerns—focusing (in order of presentation) on machine, human-machine interface, and human. The research questions were:

RQ1—Within the dominant paradigm for P300 evocation (the oddball paradigm), what alternative implementations (visual, auditory, visual/auditory, symbolic *vs.* iconic, abstract *vs.* schematic, *etc.*) yield improved performance or satisfaction in subjects?

HFE studies seek to increase reliability, availability, increase efficiency, and increase ease of use. Most literatures address various metrics of the performance for different BCI paradigms. An objective of this review is to justify those proposed interface designs in the HFE perspective, and try to identify the most promising future research directions. 

RQ2—Within the constraints of BCI instrumentation, what environmental interventions (sedentary *vs.* ambulatory implementation, comfort of instrumentation, “normality” of acquisition method, *etc.*) can yield improved performance or satisfaction in subjects?

As P300-based BCI systems increase in utility through improvements in technique and instrumentation, it is necessary to examine how they are translated into situations of actual use, where their application as assistive technologies is realized. In moving from laboratory situations to real-world use, the success of these systems (in terms of performance and uptake) will necessarily hinge on HFE.

RQ3—Guided by research conducted in other assistive technologies, how can we measure user acceptance rates? Which concepts from HFE may increase user acceptance rates of P300-based BCI systems applied in assistive technology?

Identifying accurate metrics for user acceptance is crucial for further advancement of P300-based BCI assistive technology; if such technology is to be adopted by the target audience—users with disabilities—research needs to focus on HFE of assistive technology acceptance, which consist not only of reducing errors and increasing system performance, but also of improved maintainability, reduced training requirements, reduced fatigue and physical stress, increased ease of use, increased aesthetic appearance, and overall user acceptance.

It should be stressed that, in a sense, these questions were proxies; that is, we were not directly concerned with, for example, evocation methods, or instrumentation and implementation, or specific techniques for measuring acceptance. Rather, by observing changes in the question variables, we sought to investigate the role of HFE generally. That explicit HFE terms and concepts are relatively under-represented in the literature poses challenges to a direct review of them. However, these proxy question helped to provide a framework that allowed us to conduct a systematic review (see Section 3.4.).

In that our primary motivation was to make explicit the role of HFE in P300-based BCI as an assistive technology, we had no intention to answer these questions definitively, nor exhaustively to review the body of literature. Rather, we wished to demonstrate through an exploratory review that, even in a small subset of the literature, HFE are a crucial concern at all levels of research and system design of assistive technologies, and that engaging with HFE directly can be productive in improving outcomes and satisfaction of end-users.

## 3. Review Method

We systematically searched published literature on HFE of P300-based BCI to address the following questions:

1. What signal evocation methods yield improved performance or satisfaction in subjects?
2. What environmental interventions can yield improved performance or satisfaction in subjects?
3. How can user acceptance be measured in currently available BCI technologies? Which concepts from HFE may increase user acceptance rates of P300-based BCI systems applied in assistive technology?

### 3.1. Eligibility Criteria

We included original studies that used P300-based BCI, explicitly described their signal evocation methods and interface construction, and applied multiple metrics to measure user acceptance or usability of the technology tested. Our report eligibility criteria included articles in English published within the past 27 years, since the first description of a P300-based BCI in 1988 [36].

### 3.2. Information Sources

The following online databases were searched:

- Compendex, to provide an engineering perspective;
- IEEExplore, to provide a targeted electrical/electronics engineering perspective;
- MEDLINE, to provide a medical perspective; and
- Web of Science, to provide a broad-spectrum perspective.

We constrained our search to the time period since the first description of a P300-based BCI, knowing that most research activity on the topic has only occurred in recent years, and to studies published in English. The search period lasted two months, and the final search was conducted on 30 March 2015.

### 3.3. Search

The general search strategy included key terms such as “BCI,” “P300,” “interface.” In addition, the search was expanded with specific terms to address the research questions; for example, “oddball paradigm” term for RQ1, “signal acquisition” for RQ2 and “user acceptance model AND assistive technology” for RQ3. The main search constraint encountered was the relatively small body of literature pertinent to HFE of P300-based BCIs.

### 3.4. Study Selection

Search inclusion and exclusion criteria were based on number of research subjects, oddball implementation methods, control groups or comparison groups, and measures of user satisfaction, described below. The literature on P300-based BCI is broad. However, much of it is concerned with the technical aspects of improving signal evocation, acquisition, and interpretation. This is fundamental work, but our research questions directed us towards studies of application. Thus, any review study needed to have subjects, that is, a non-zero population. As our concern is assistive applications, we screened for studies with representative subjects (*i.e.*, subjects with neuromuscular disabilities and assistive needs). However, as this group is a small percentage of the total population, we consistently faced a “small *n*” problem, and thus did not exclude useful studies if they included only healthy participants. We excluded no study on the basis of subject age, gender, or specific neuromuscular disability.

Following RQ1, we screened for studies that demonstrated or compared alternative implementations of the oddball paradigm to evoke the P300 wave. The final selection of studies for RQ1 also needed to present a variety of alternative implementations. Following RQ2, we screened for studies that demonstrated or compared alternative BCI implementation environments. The final selection of studies for RQ2 also needed to present a variety of alternative implementation environments. We screened for studies with a design that included a control group or that managed treatments so as to provide a functional control comparison. Given the nature of BCI research on assistive technologies, there were rarely true controls. Most often, we selected studies that compared treatments or levels of a given treatment, or both. However, we did not exclude studies that sought to demonstrate a new assistive application, if such study informed our research questions. Following RQ3, we screened for studies that accounted for user satisfaction or acceptance among its outcome metrics. For studies that did not explicitly include such a component, we screened for those studies the outcomes of which could be logically linked with user acceptance (improved performance in simulated life tasks, increased comfort, virtual or actual self-directed mobility, *etc.*).

**Figure 1:**
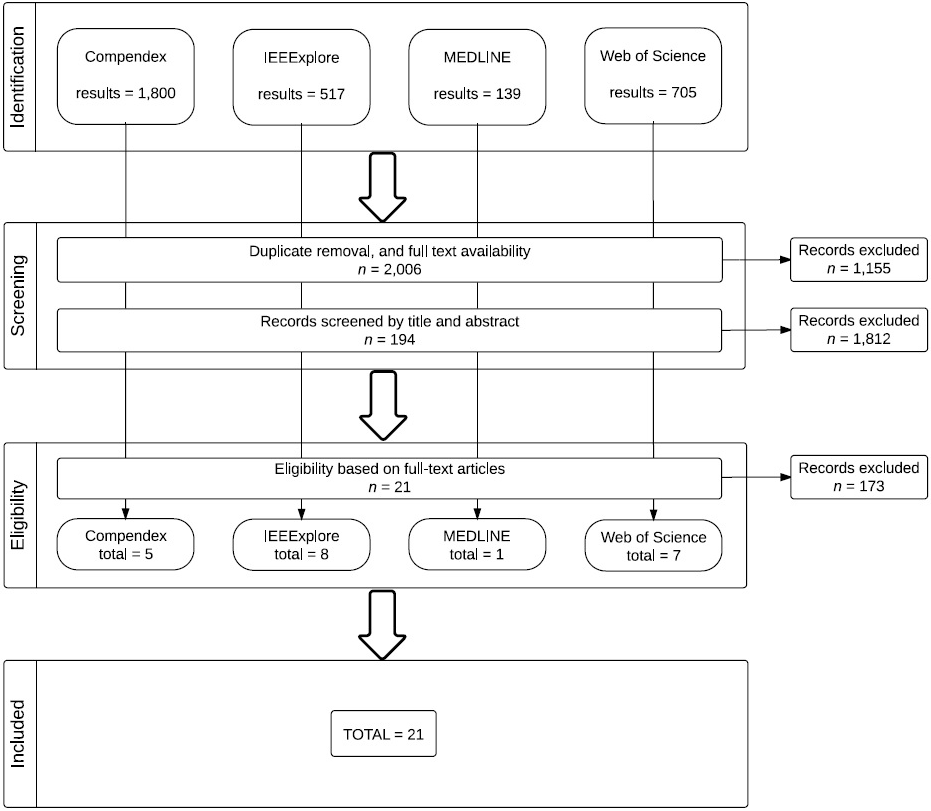
PRISMA flow diagram of this review.

Given the nature of BCI research on assistive technologies, there is a narrow range of study designs employed. Most frequently we encountered small-*n*, within-subject designs. That said, we did not screen for design type, beyond the requirement already stated that the design involve human subjects. To guide the review process, the authors followed the Preferred Reporting Items for Systematic reviews and Meta-Analyses (PRISMA) approach. PRISMA makes use of a 27-item checklist and a four-phase process to developed to improve systematic reviews and transparent reporting of such reviews [42]. The PRISMA flow diagram of the study selection process is presented in Figure 1.

### 3.5. Data Items

Information was extracted from each included article to focus on the following variables:

- Population: Initially, we screened for studies with representative subjects (*i.e.*, subjects with neuromuscular disabilities and assistive needs), but, due to the small-*n* problem, we decided not to exclude useful studies if they included only healthy participants. We excluded no study on the basis of subject age, gender, or specific neuromuscular disability.
- Interventions: For RQ1, we screened for studies that demonstrated or compared alternative implementations of the oddball paradigm to evoke the P300 wave. For RQ2, we screened for studies that demonstrated or compared alternative BCI implementation environments. For RQ3, we screened for studies that measured usability and/or user acceptance of assistive technologies using BCI.
- Comparators: We screened for studies with a design that included a control group or that managed treatments so as to provide a functional control comparison. Given the nature of BCI research on assistive technologies, there were rarely true controls. Most often, we selected studies that compared treatments or levels of a given treatment, or both. However, we did not exclude studies that sought to demonstrate a new assistive application, if such study informed our research questions.
- Outcomes: We screened for studies that accounted for user satisfaction or acceptance among its outcome metrics. For studies that did not explicitly include such a component, we screened for those studies the outcomes of which could be logically linked with user acceptance (improved performance in simulated life tasks, increased comfort, virtual or actual self-directed mobility, *etc.*)
- Study designs: Given the nature of BCI research on assistive technologies, there is a narrow range of study designs employed. Most frequently we encountered small-*n*, within-subject designs. That said, we did not screen for design type, beyond the requirement already stated that the design involve human subjects.

## 4. Review Results

### 4.1. Study Selection

A total of 21 studies were identified for inclusion in the review. The search of these databases yielded 3161 citations. Of these citations, we excluded 1155 citations because they were duplicates, because the studies were not in our target language (English), or because we could not locate the full text of the study. Of the 2006 remaining citations, we used software-based term screening to identify studies that would inform our research questions. Through this process, we excluded a further 1812 citations. The remaining 194 articles were reviewed by title and abstract to select those studies most appropriate to the review. A final total of 21 articles were selected—five from Compendex, eight from IEEExplore, one from MEDLINE, and seven from Web of Science. 

### 4.2. Study Summaries

Table 1, Table 2 and Table 3 list the 21 articles reviewed. Entries marked as “N/A” were not applicable to the study in question. This is to be distinguished from the “Unknown” entry, which indicates that information was not explicitly stated. Detailed explanation and analysis are provided in later sections.

**Table 1:** Studies selected for RQ1.

	Salvaris and Sepulveda (2009) [43]	Townsend *et al.* (2010) [44]	Acqualagna *et al.* (2010) [45]	Aloise *et al.* (2012) [46]
Aim	To study the effect of changes to the visual aspects of oddball protocol, e.g., dimensions of the symbols, distance between the symbols, and colors	To compare checkerboard paradigm with the conventional row and column paradigm	To test a novel rapid serial visual presentation paradigm in an offline study, investigating two different presentation speeds and two different color conditions	To study and compare performances of gaze-independent P300-based BCI and the conventional P300 speller interface
Study design	Within-subject	Within-subject	Within-subject	Within-subject
Participants	8	18	9	10
Age	Mean 22.3, 19–28	Undergraduate students	24–31	Mean 26.82, σ = 4.21
Gender ratio (male:female)	6:2	11:7	8:1	6:4
Disabled participants	0	3	0	0
Type of non-healthy participant	N/A	ALS	N/A	N/A
Signal evocation method	Conventional oddball (6 × 6 matrix)	8 × 9 matrix	Stimuli presented in the center of the screen on a gray background, with a height of 3.5 cm or 1° visual angle	Each character belonged to two sets of size *n*. Each set presented at the center of screen, forming a regular geometric figure, with characters displayed at the vertices
Interface construction	EEG with Biosemi ActiveTwo system at a sampling rate of 512 Hz; 66 channels were used, including 2 as reference; out of the 64, 8 were selected according to performance Participants sat 1 m from a 19 inch TFT screen	EGG with a standard 32-channel electrode cap; amplified and digitized by two g.tex 16-channel USB biosignal amplifiers; ALS users sat in their wheelchairs Participant sat 1 m from a computer monitor	EEG with a 64-channel actiCAP system; EEG data were sampled at a rate of 1000 Hz and subjected to off-line analysis Participants sat 80 cm from a computer monitor	Scalp EEG potentials were measured using 10–10 standard on an elastic cap; the EEG was acquired using a g.USBamp amplifier, sampled at 256 Hz Eye movements were monitored on an eye tracker system
Environment	Laboratory	Laboratory	Laboratory	Laboratory
Acceptance measure	N/A	N/A	N/A	NASA workload metrics; User acceptance score (subjective)
	**Akram *et al.* (2013) [47]**	**Pathirage *et al.* (2013) [48]**	**Alcaide-Aguirre and Huggins *et al.* (2014) [49]**	**Ma *et al.* (2014) [50]**
Aim	To study a modified T9 (text on 9 keys) interface with word suggestions	To test an oddball-based paradigm with a visual interface to conduct daily living tasks; subjects could control a robotic arm mounted on wheelchair to grasp items under commands through the BCI	To evaluate a new P300 interface with reduced amount of information through series of experiments on performing hold-release and deactivation tasks	To study a hybrid P300-based EEG interface integrated with eye movement recognition, using both EEG and electrooculography (EOG), in performing two tasks of controlling robots
Study design	Within-subject	Proof of concept	Within-subject	Within-subject
Participants	10	6	7	13
Age	N/A	24–40	14–40	22–30
Gender ratio (male:female)	10:0	5:1	5:2	11:2
Disabled participants	0	0	0	0
Type of non-healthy participant	N/A	N/A	N/A	N/A
Signal evocation method	T9 interface	5 × 5 non-uniform grid was constructed with algorithms, thus the oddball paradigm could be applied onto 25 points	5 × 6 matrix for a P300 speller; two layouts were used, in one of which numbers were replaced with asterisks to reduce perceptual errors	Conventional oddball matrix interface for EEG evocation
Interface construction	EEG data was acquired through a 32-channel BrainAMP MR amplifier with a sampling frequency of 250 Hz; EEG potentials were measured using 10–20 standard	8-channel electrode cap, a g-tec USBAmp-8 biological signal amplifier, the input signal was sampled at 256 Hz	16-channel EEG electrode cap; subjects sat in front of a computer screen with BCI layout	EEG signal was recorded with 8 electrodes with a sampling rate of 256 Hz and was downsampled to 64 Hz Three event-related potential components were exploited: VPP, N170, and P300; P300 was evoked by oddball event, while the other two were evoked by the configural processing of facial image
Environment	Laboratory	Laboratory	Laboratory	Laboratory
Acceptance measure	N/A	N/A	N/A	N/A

**Table 2:** Studies selected for RQ2.

	Klobassa *et al.* (2009) [51]	Escolano *et al.* (2010) [52]	Castermans *et al.* (2011) [53]	Li *et al.* (2011) [34]
Aim	To investigate auditory operation of P300 speller	To report a BCI telepresence system and to study its applicability to ALS patients	To investigate the use of P300-based BCI in an ambulatory condition	To evaluate contributing factors to user performance in BCI applications
Study design	Two-group, with partial control (mixed design: one group had constant treatment, while other group had treatment gradually reduced)	Proof of concept	Within-subject (each subject performing at three treatment levels)	Within-subject (two treatments at three levels of treatment)
Participants	10	1	7	20
Age	Mean 47, 22–68	54	25–33	Healthy (mean 27.9, σ = 3.6) Disabled (mean 43.7, σ = 14.8)
Gender ratio (male:female)	6:4	Unknown	Unknown	14:6
Disabled participants	0	1	0	10
Type of disability	N/A	ALS	N/A	Various neuromuscular disabilities
Signal evocation method	Auditory and auditory/visual oddball Standard speller	Visual (iconic) oddball	Quasi-oddball (target not rare) Standard speller	ABC and Frequency oddball Standard speller
Interface construction	EEG cap, reclining chair	EEG, audio-visual interface over Internet	EEG cap, accelerometer strapped to head, lower limb kinematics, recorded with infrared cameras	EEG cap, each method tested in 3 screen sizes (monitor, GPS, and phone) at appropriate distance
Environment	Laboratory	Patient at home (Germany) Telepresence robot (Spain)	Laboratory Quasi-ambulatory (subject on treadmill)	Laboratory sedentary
Acceptance measure	N/A	N/A	N/A	N/A
	**Duvinage *et al.* (2013) [54]**	**Tai *et al.* (2013) [55]**	**Corralejo *et al.* (2014) [56]**	**Riccio *et al.* (2015) [57]**
Aim	To compare medical-grade and consumer-grade EEG BCI by comparing performance in P300	To propose a BCI to enable disabled individuals to utilize the Internet	To develop and assess an assistive tool for operating electronic devices at home by means of P300-based BCI	To evaluate hybrid P300-based BCI with electromyographic error correction
Study design	Within-subject (4 crossed treatments: consumer *vs.* medical, sitting *vs.* treadmill)	Proof of concept	Proof of concept	Proof of concept, and within-subject (2 treatments: P300 control and P300 w/correction)
Participants	9	14	15	11
Age	24–34	20–25	Mean 50.27, 35–68	Healthy (28 ± 7) Disabled (48, 54, 49)
Gender ratio (male:female)	8:1	Unknown	7:8	Healthy (5:3) Disabled (2:1)
Disabled participants	0	0	15	3
Type of disability	N/A	N/A	Varied motor and cognitive disabilities	Severe motor disabilities
Signal evocation method	Visual oddball	Visual oddball	P3Speller-based Visual (iconic) Matrix menu based	Visual (color and shape)
Interface construction	Emotiv Epoc (8 channel) ANT (used at same positions)	Not explicit	EEG	EEG and EEG+EMG
Environment	Laboratory sedentary and ambulatory	Not explicit Laboratory	Laboratory	Laboratory
Acceptance measure	N/A	N/A	Questionnaire	Interview

**Table 3:** Studies selected for RQ3.

	**Lightbody *et al.* (2010) [58]**	**Riccio *et al.* (2011) [59]**	**Holz *et al.* (2013) [60]**
Aim	To develop a BCI system that is customizable in terms of its technology and applications	To evaluate usability through subject performance, workload, and satisfaction and to compare two applications for P300-based BCI	To evaluate usability of new SMR-BCI-controlled gaming prototype based on effectiveness, efficiency, and user satisfaction
Study design	Proof of concept	Within-subject	Within-subject
Study description	Quantitative and qualitative research of user requirements in a 3-year iterative development; SSVP, ERD/ERD, and P300 paradigms; six 30-minute sessions with 4 oscillatory visual stimuli (30, 35, 40, and 45 Hz) presented simultaneously	2 conditions: split—oddball screen to select command and application screen where commands were executed; overlaid—oddball paradigm overlaid the application; 3 tasks: Internet browsing, word processing, and software configuration	6 sessions of BCI interactions with Connect-Four, strategic game with two competitive players; 2 types of tasks: copy task and free playing mode
Participants	20	8	4
Age	Unknown	26.7 ± 1.3 years	45–48
Gender ratio (male:female)	Unknown	6:2	Unknown
Disabled participants	5	0	4
Type of non-healthy participant	Varied motor and cognitive disabilities	N/A	Severe motor disabilities; 2 out of 4 were locked-in
Signal evocation method	Visual (stimuli rendered through LEDs on the outer edges of the screen)	Visual	Visual
Interface construction	EEG; Mightex-universal LED controller; TMSi Porti amplifier at a sampling rate of 2048 Hz	EEG; g.USBamp. amplifier; BCI2000 brain transducer	EEG; 2-class SMR-BCI game; Brain Vision and g.USBamp. amplifiers;
Environment	Laboratory	Laboratory	Laboratory
Acceptance measure	Workshops, questionnaire, and focus group	NASA Task Load Index (TLX) questionnaire for subjective workload, visual analogue scale for user satisfaction, and unstructured interview	NASA TLX for subjective workload; visual analogue scale for overall satisfaction; Extended QUEST and ATD-PA for satisfaction regarding different dimensions of BCI device; semi-structured interview and focus group
	**Bonnet *et al.* (2013) [61]**		**Zickler *et al.* (2013) [62]**
Aim	To design and implement a multi-user BCI-based gaming system and to qualify and quantify the influence of multi-user paradigm on BCI interaction		To evaluate usability of the Brain Painting prototype according to the standards of the International Organization for Standardization.
Study design	Within-subject		Within-subject
Study description	Brain Arena, simple football video game based on hand motor imagery (MI), evaluated based on 3 paradigms (solo, collaborative, and competitive) in 2 experiments; classification accuracy = performance metric		3 tasks: copy spelling, copy painting, and free painting; 2 monitors for painting: BCI command screen and “canvas”; recalibration available to support accuracy of at least 80%
Participants	20		4
Age	23–52		39–55
Gender ratio (male:female)	15:5		Unknown
Disabled participants	Unknown		4
Type of non-healthy participant	Unknown		Varied motor and cognitive disabilities
Signal evocation method	Visual		Visual
Interface construction	EEG; USBAmp amplifiers; GAMMA Caps with 16 active electrodes; OpenVibe software platform; Ogre 3D rendering engine		EEG; 6x8 matrix for P300 spelling and Brain Painting app; g.USBamp. amplifier; BCI2000 software; Brain Vision Analyzer 2
Environment	Laboratory		Laboratory and field (at home with external distractions)
Acceptance measure	Questionnaire (Likert scale, open questions)		Accuracy to measure effectiveness; ITR and utility metric for efficiency; NASA TLX for subjective workload; QUEST and ATD PA for user satisfaction; semi-structured interview

### 4.3. Study Characteristics

#### 4.3.1. Participants

Most of the BCI-related studies aimed at providing a tool through which people could communicate with others or give control instructions to machines without muscular input. Different BCI paradigms were used and tested among small groups of participants. For different individuals, the system would have to be tuned according to their unique characteristics. Ideally, for the purpose of practical usage, researchers need to consider having their experiment conducted on similar groups of potential users, with a sufficiently large sample size.

Among the studies that we reviewed, the average number of participants was 10.7, with a maximum of 20 and a minimum of 1. Some studies engaged a very small group of subjects because their studies only focused on proof of concept [52], or because all their subjects were disabled [60,62]. The age of participants varied by study. Nineteen out of twenty-two articles explicitly disclosed the age information of their subjects; one article only mentioned that the participants were undergraduate students [44]; two did not mention participants’ age [47,58]. Based on the known information, the oldest participant was 68 years old, while the youngest one was 14. Generally, people with disabilities tend to be older and people in the healthy group tend to be younger. Sixteen articles reported the gender distribution of their participants [34,43,44,45,46,47,48,49,50,51,53,54,56,57,59,61]. The average percentage of male subjects among these papers was 76.4%. We observed that, in all experiments with gender information, the male population was predominant in the participant pool. Eight studies recruited participants with disabilities [34,44,52,56,57,58,60,62]. The most common type of disability, as seen in Escolano *et al.* 2010 [52] and Townsend *et al.* 2010 [44], was ALS. Other articles did not explicitly list the disability type, but described participants’ disabilities as “varied” or “severe” [34,50,56,57,58,60,62]. In addition, three articles mentioned “cognitive disabilities” [56,58,62]. Twelve studies reported all healthy subjects [43,45,46,47,48,49,50,51,53,54,55,59], and one study did not disclose this information [61].

#### 4.3.2. Experimental Design

With the relatively small sample sizes, most of researches used within-subject designs for their experiments. Each participant would have opportunity to conduct all possible configurations of different BCI paradigms. Five studies, however, did not make comparisons, but only conducted proof of concept in their experiments [48,52,55,56,58].

#### 4.3.3. Study Environment

Most of the experiments took place in a laboratory environment. Only two studies utilized a “daily life” environment, for example in the home [52,62]. In almost all studies, participants were in a sedentary position. Only two studies explored ambulatory conditions [53,54].

### 4.4. Results of Individual Studies

#### 4.4.1. Studies Selected for RQ1

Research Question 1 concerned approaches to P300 evocation that benefitted HFE. Although the first version of the P300-based BCI was proposed nearly 30 years ago, the general understanding of BCI designs is still evolving rapidly. The conventional matrix (row- and column-based) oddball paradigm is a productive approach, but it has limitations. The throughput rate is relatively low, certain training is required, and its reliance on eye movement would limit its usability for people with certain disabilities. In addition, users may favor different types of configurations. A good design, in the HFE perspective, should increase reliability, reduce training requirements, reduce loss of time, and improve system performance. For these reasons we investigated various evocation designs and their impact on various HFE metrics, e.g., training requirements, effectiveness, and efficiency.

With regard to the design of stimulus presentation, we observed three research directions: (1) improvement on variations of the conventional matrix-based oddball paradigm [43,44,48,49]; (2) non-matrix variations [45,47]; and (3) hybrid interfaces that utilized more than one feedback signal [50,51,57].

**Figure 2:**
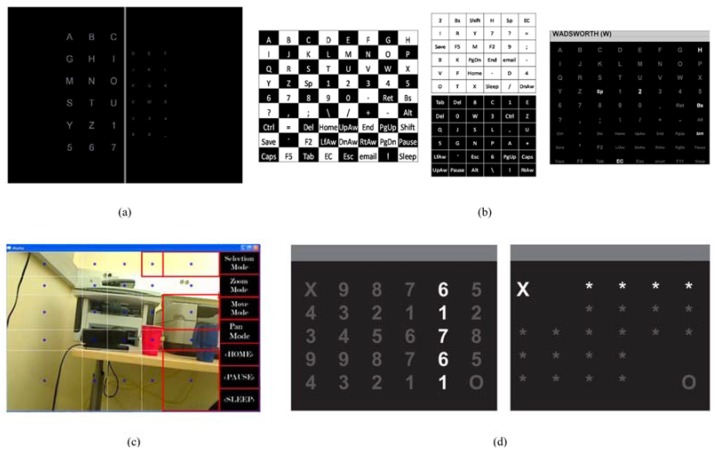
(**a**) An example of size differentiation of fonts in Salvaris and Sepulveda (2009) [43]; (**b**) The checkerboard approach of Townsend *et al.* (2010) [44]; (**c**) The non-uniform grid approach of Pathirage *et al.* (2013) [48]; (**d**) The two-layouts approach of Alcaide-Aguirre and Huggins (2014) [49], where the left layout served for “holding” usage.

Salvaris and Sepulveda (2009) [43] studied the effects of different visual aspects on the conventional P300-based oddball protocol, as shown in Figure 2a. Specifically, three factors were considered: the dimensions of symbols, the distance between symbols, and the color of symbols. They did not find a single configuration that outperformed all others across all participants. However, they found that a white background gave the best performance, while small symbol size gave the worst.

**Figure 3:**
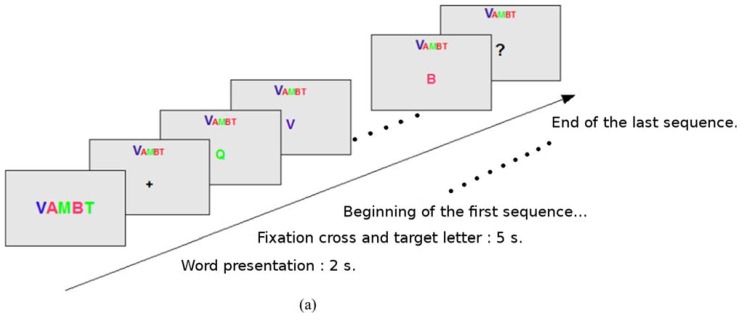

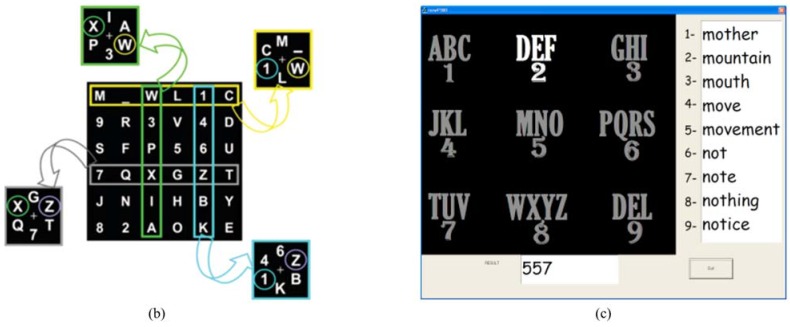
(**a**) The rapid serial visual presentation approach developed by Acqualagna *et al.* (2010) [45] (image adapted); (**b**) Grouped presentation of Aloise *et al.* (2012) [46]; (**c**) The T9 paradigm of Akram *et al.* (2014) [47].

Townsend *et al.* (2010) [44] compared a proposed checkerboard presentation with the conventional row and column presentation. An 8 × 9 black and white checkerboard matrix was developed, as in Figure 2b. They found that the proposed design gave significant improvement in terms of mean online accuracy and mean bit rate. Experimental results showed that the improvement for ALS participants was more pronounced than that for non-clinical participants. Pathirage *et al.* (2013) [48] explored a new usage for P300-based BCIs. They implemented an oddball-based paradigm with a visual interface to control a robotic arm mounted on a wheelchair to grasp designated items. A camera imaged the surrounding environment and then, using a computer vision algorithm, a grid of dots was overlaid on the photo, as shown in Figure 2c. These dots were like the conventional letters in a row and column P300 speller. Participant could trigger the control command through oddball-based evocation and instruct the robotic arm to complete the task. A 5 × 5 non-uniform grid was constructed, which could be regarded as a variation of the common row and column setup. Alcaide-Aguirre and Huggins (2014) [49] took another direction with a hold-release task with BCI. Instead of just triggering one control command or typing one letter, their approach used two layouts that were capable of activating a “hold” instruction. With a 5 × 6 matrix, the first layout was similar to the original, while the information on the second layout was reduced, so that participants could have more focus on certain icons and “hold” them, as shown in Figure 2d. Acqualagna *et al.* (2010) [45] developed a rapid serial visual presentation approach that featured a sequence of letters rapidly presented at the center of the screen following the oddball pattern, as shown in Figure 3a. This paradigm did not require eye movement of participants, which is promising for patients with oculomotor impairment. In the study, two color setups and two speed setups were tested across all subjects. They found that, although there was no significant effect of color, slower symbol presentation gave higher accuracy.

Aloise *et al.* (2012) [46] proposed a gaze-independent P300-based BCI called GeoSpell. Instead of presenting all letters in a matrix, as in the conventional matrix approach, they grouped all letters into several subsets of size *n*, so that each letter belonged to two subsets, as shown in Figure 3b. During each trial, a subset of letters would form a regular geometric figure at the center of the screen, with characters displayed at the vertices (notice the hexagonal arrangement in Figure 3b). The online accuracy and speed for GeoSpell was significantly lower than that of the conventional P300 speller, but certain users may still favor GeoSpell as it does not require gaze shift. Akram *et al.* (2014) [47] demonstrated a phone keyboard, similar to a text on nine keys (T9) approach, shown in Figure 3c, which took advantage of initial typing and word suggestion. The layout of the presentation was a 3 × 3 matrix where each entry contained more than one symbol. Participants could choose from the nine possible entries and form words with completion suggestions. Compared to the conventional matrix approach, this proposed scheme decreased typing time by 51.87%, which shows its efficiency. In Ma *et al.* (2014) [50], a hybrid interface was proposed, taking advantage of both EEG and electrooculography (EOG). A P300-based EEG interface was integrated with an eye movement tracking interface, and two tasks were performed. The EEG evocation presentation had eight arrow icons around the center of the screen, pointing in eight directions. Inverted facial images were used as stimuli and were placed upon the arrow icons to trigger the EEG signal. This approach had a significant positive effect on the efficiency of the control process. 

#### 4.4.2. Studies Selected for RQ2

Research Question 2 concerned environmental interventions in P300-based BCI systems that could have a positive impact on the HFE of performance and uptake. As research in these systems begins to move more and more beyond the laboratory, attention to HFE-relevant system implementations will be crucial to future development, and the eventual feasibility of P300-based BCI for assistive use [41].

The connection of these studies to HFE is evident when one considers the impact on the cognitive-affective responses and physical needs of users. The latter point is more readily evident, as questions of voluntary mobility and motion are paramount for many disabled individuals. The former point, that of cognitive-affective response, is not as readily evident, but is necessarily a crucial factor in performance. Assistive technologies that serve to aid users in real-world tasks and in real-world environments are more than assistive, they are enabling. That is to say, positive affective response to being enabled could synergistically lessen cognitive load, or at least make cognitive loads more easily tolerated. For this reason, studies that relate to “normality” (of environment, of task, of simplicity, of autonomy) directly speak to the HFE of BCI systems.

Castermans *et al.* (2011) [53] investigated the implementation of P300-based BCI in ambulatory conditions. The BCI was augmented by accelerometer and kinematic recording in order to correct for gait noise. They concluded that ambulatory P300-based BCI was feasible, but that noise correction did not improve significantly over raw BCI data. To compromise between portability and performance, they proposed the use of the xDawn spatial filter with an EEG configuration over the occipital and parietal areas of the brain. In a similar study, Riccio *et al.* (2015) [57] investigated the use of EMG signals for BCI error correction, as such a method could be of benefit to subjects with residual muscle activity. Although the study was a standard laboratory implementation of a P300-based BCI, the utility of a channel reading muscular activity could be extrapolated to real-world tasks and environments. They found that while the hybrid system (EEG and EMG) had lower throughput, it provided increased accuracy that allowed for decreased overall task time, and reduced user workload and frustration. They concluded that the hybrid BCI is a significant step in developing functional (*i.e.*, real-world) systems. Corralejo *et al.* (2014) [56] demonstrated an implementation of P300-based BCI to enable the operation of electronic devices in the home by people with severe impairment. They tested a P3Speller-based interface that displayed menus to guide control devices that were then directly operated by means of infrared emitter. In general, the researchers were impressed with the results obtained, given the simulation of a real-life task environment, and given the range of cognitive and motor impairments of the subjects. They concluded that degree of impairment was not a relevant issue in operation of the tool, and suggested that it could be easily modified to suit the needs and requirements of users, and would decrease dependence on caregivers. Duvinage *et al.* (2013) [54] compared medical-grade *vs.* consumer-grade EEG-based BCI for P300 applications. While the consumer-grade product had much lower cost and was simpler to operate, they found that its performance was much worse and its relative costs much higher than the medical-grade BCI. They concluded that the consumer-grade system was appropriate for non-critical applications such as gaming and communication, but for critical applications such as rehabilitation and prosthesis control the medical-grade BCI was the necessary choice. They suggested that a low-cost BCI for critical applications should be developed.

Escolano *et al.* (2010) [52] demonstrated a telepresence system, whereby an impaired subject could achieve virtual mobility through BCI control of a robot that provided audiovisual feedback and environmental interaction. They concluded that their system was advantageous in that it did not require sustained attention (as the user decided the action of the robot, but the robot autonomously executed the command); it had rehabilitative potential for ALS patients by allowing spatial, decision-making, and communicative mental exercise; and—unusually for BCI systems—it was considered a fun way for impaired subjects to engage in brain training (as reported by the subjects themselves). Klobassa *et al.* (2009) [51] investigated auditory control of a P300-based BCI, to enable use by subjects that may have visual or oculomotor impairment, thus rendering visual evocation unacceptable. They tested environmental sounds, as opposed to abstract or “inhuman” sounds. The goal was not to compare evocation methods, as such, but rather to see if evocation methods could be adapted to the environmental and physical needs of users, and still offer information rates high enough for useful communication. They found that an auditory system had lower performance than a visual system, and suggested that this was related to the relative complexity of the task as sight allowed for effective filtering of input, whereas this was more difficult with hearing. They concluded that the results were nevertheless promising, and that further development of auditory systems was worthwhile. Li *et al.* (2011) [34] investigated the impact of environmental variables of BCI system design. In addition to comparing interface types (ABC *vs.* frequency-based matrices), they simulated real-life use by adapting the system to common screen types (monitor, navigation device, cellphone). In terms of the interface construction, they found that screen size had a significant effect on user performance and preference. They concluded that this fact should inform future development of real-world assistive systems. Tai *et al.* (2013) [55] demonstrated a P300-based BCI system designed to allow use of the Internet by severely disabled individuals. The design integrated a visual evocation mechanism incorporated into a specially constructed web page. The system allowed for cursor control and page navigation, activities comparable to real-world use of the Internet for daily tasks. They demonstrated that a BCI could be integrated into a standard computer navigation element (the cursor), and that this system could be integrated into a web browser. They concluded that the simplicity and intuitiveness of the system allowed for high accuracy to be attained quickly, and that future development would allow for impaired subjects to browse the Internet autonomously.

#### 4.4.3. Studies Selected for RQ3

Research Question 3 addressed the issue of user acceptance of BCI systems. These studies used metrics with varying degrees of complexity to capture objective and subjective data regarding user acceptance. For example, while Bonnet *et al.* (2013) [61] used a questionnaire with Likert scale and open-ended questions, Holz *et al.* (2013) [60] used the NASA Task Load Index (TLX) [63] to measure participants’ subjective workload, visual analogue scale questionnaires for overall satisfaction, the Extended Quebec User Evaluation of Satisfaction with assistive Technology (Extended QUEST [64]) and the Assistive Technology Device Predisposition Assessment (ATD-PA [65]) for satisfaction regarding different dimensions of the BCI device, and semi-structured interviews and focus groups to gather qualitative data.

User acceptance is related to the human–computer interaction concepts of the gulf of execution, *i.e.*, translating goals to actions, and the gulf of evaluation, *i.e.*, understanding the machine output [66]. User acceptance metrics focus on better understanding the factors that play into the human perception of the affordances and capabilities of a system before engaging with it. For this review, we looked at the factors associated with the interface construction, which could be used to bridge the gulf of execution. The review of various P300 signal evocation methods to identify the optimal system feedback method, which would bridge the gulf of evaluation, helped us better understand the connection to user acceptance rates. 

Bonnet *et al.* (2013) [61] evaluated the appropriate levels of feedback design in a BCI video game and the gameplay multi-user paradigm by examining participants’ performance and subjective experience of engaging with the system. To address the HFE impacting user acceptance level, the researchers used a hand motor imagery (MI)-based football video game, with the purpose of identifying the balance between immersion and simplicity for the feedback design. They conducted a two-step experiment to compare subject performance in single-user *vs.* multi-user (collaborative) BCI interaction in gameplay, and then two approaches in a multi-user setting (collaborative and competitive). In the single-user condition, the BCI interaction with the game environment presented a closed-loop system, and in multi-user condition, the system could connect the users at four different levels of the loop (brain signal processing, decision-making, interaction technique, and application). The researchers concluded that while mean classification performance and qualitative questionnaire results were in favor of the collaborative BCI interaction, one third of participants found collaborative feedback distracting. 

Lightbody *et al.* (2010) [58] conducted a study to improve a design of an integrated BCI system, using the high-frequency Steady State Visual Evoked Potential (HF-SSVEP) approach. The objectives from the HFE perspective included to increase the economy of production (economy of effort in this case), reduce training requirements, improve system performance, maintainability, and reliability, and to increase the user acceptance levels. User-centered design and development of the system entailed engaging two user groups (healthy and disabled subjects) as well as a lead user, who participated in prototype testing and provided feedback in early development stages of an iterative development process. The workshops and questionnaires conducted in this study provided researchers with technical user requirements, such as integration of access to multimedia and development of interoperable systems, and general recommendations to improve training techniques and the usability of intuitive graphical user interfaces (IGUIs).

Riccio *et al.* (2011) [59] compared two P300-based BCI applications evaluating their usability in terms of effectiveness, measuring accuracy, efficiency, measuring subjective workload, and satisfaction, using visual analogue scales and unstructured interviews. Using such a comprehensive approach to measuring usability, the researchers collected data that addresses multiple HFE objectives, such as improving system performance while reducing the operator fatigue and stress, addressing aesthetic appearance, increasing ease of use, and reducing training requirements. The authors evoked the P300 signal under two conditions: two separate screens for oddball presentation of command and execution, and one screen with oddball paradigm overlaying the application, using three different tasks—Internet browsing, word processing, and configuration of the software. Contrary to their expectations, the researchers did not observe any significant difference in effectiveness, efficiency, and user satisfaction between the overlaid and split conditions.

Holz *et al.* (2013) [60] evaluated usability of an sensorimotor rhythm (SMR)-based BCI gaming application design. Similarly to the study conducted by Riccio *et al.* (2011) [59], the focus was on HFE of system design that would yield high user acceptance levels by improving system performance while reducing operator fatigue and stress, addressing aesthetic appearance, increasing ease of use, and reducing training requirements. The application’s usability was evaluated in accordance with ISO 9241-210 [67] measuring effectiveness (accuracy), efficiency (information transfer rate and subjective workload), and satisfaction (visual analogue scale, Extended QUEST, ATD-PA, semi-structured interview, and a focus group discussion). Each study subject participated in nine sessions, five of which were BCI operations, consisting of copy-task and free modes. The researchers found that overall usability metrics of SMR-based BCI were lower than those of P300-based BCI (as reported in other studies): effectiveness of three (out of four) users was low to medium, efficiency (mental workload) was moderate, and while users rated their satisfaction relatively high regarding weight, safety, and learnability of the device, they were dissatisfied with its dimensions, adjustment, ease of use, and speed.

Zickler *et al.* (2013) [52] engaged four subjects with varied motor and cognitive disabilities to evaluate the usability of the P300-based Brain Painting prototype measuring its effectiveness (accuracy), efficiency (information transfer rate, utility metric, and subjective workload), and user satisfaction (QUEST, ATD PA, and Device Form). Subjects navigated a test display with a 6 × 8 matrix to perform the tasks of copy spelling, copy painting, and free painting. At the beginning of each task, subjects copy-spelled a word to assure a signal rendering accuracy of at least 80%. The study design had the basic operational objectives of HFE embedded in the task conditions, so the feedback collected from the study participants was focused primarily on reliability, maintainability, availability and integrated logistic support, user perception of the system (gulf of evaluation) and their objectives. High external validity of the experiment was granted by the test setting, either at the subject’s home or at the Information Center for Supported Communication in Germany, which deliberately did not eliminate background noise or minor distractions. The study results demonstrated high overall levels effectiveness, efficiency, and user satisfaction, while providing an extensive list of user requirements for both hardware and software.

Overall, the key user acceptance metrics applied to evaluate P300-based BCI systems included effectiveness, efficiency, user satisfaction, subjective workload, and ease of learning how to use the system.

The most commonly used measure for user acceptance, applied in all five studies reviewed, was a questionnaire of varying type and degree of sophistication. For instance, Bonnet *et al.* (2013) [61] used a seven-point Likert scale questionnaire and a questionnaire with open-ended questions, which yielded qualitative data with subjective user impressions; while other researchers (Holz *et al.* (2013) [60] and Zickler *et al.* (2013) [62]) evaluated their BCI system’s user acceptance in accordance with ISO 9241-210, measuring accuracy in performing tasks, information transfer rate, subjective workload, and qualitative data using questionnaires with a visual analogue scale, Extended QUEST, ATD-PA, semi-structured interviews, and focus group discussions.

## 5. Discussion

### 5.1. Summary of Evidence

#### 5.1.1. Evidence for RQ1

Research Question 1 was “Within the dominant paradigm for P300 evocation (the oddball paradigm), what alternative implementations (visual, auditory, visual/auditory, symbolic *vs.* iconic, abstract *vs.* schematic, *etc.*) yield improved performance or satisfaction in subjects?” This question concerns several objectives of HFE, that is to increase system performance, reduce loss of time, increase efficiency and reliability, and increase ease of use. A good design of the BCI stimulation presentation would much benefit its end-users for efficiently conducting various tasks, e.g., communicating with other people and controlling machines and robots, with minimized effort and reduced error. Out of the eight articles reviewed to address RQ1, three [43,44,47] directly compared the performance of a new evocation approach with the conventional row and column matrix first proposed by Farwell and Donchin (1988) [36], two [48,50] extended the usage of P300-based BCI by integrating computer vision and a robotic control interface or integrating the EOG scheme as assistance for robot control, and three [45,46,57] proposed new paradigms for specific usages, e.g., gaze-independent interfaces, and “hold and release” tasks. We were mainly interested in how evocation stimuli were presented to participants and how this affects HFE metrics. As mentioned in preceding sections, researchers focused on extending and improving the conventional row and column based approach in three main directions: optimizing the layout parameters of the conventional paradigm, developing new layouts of letters and icons that better serve users, and integration of other assistive technologies. There is potential for improving on the oddball paradigm by adjusting its configurations, e.g., font size, color choice, distance between symbols, and arrangement of matrix entries (uniform or non-uniform). Several studies only focused on a proof of concept, and there was no comparison in terms of quantitative HFE metrics. Certainly, extending the usability of BCI into more task situations can be regarded as an improvement in HFE, as it might benefit certain groups of people with disabilities and enable them with more control/communication choices. However, there are no universal rules of design in the construction of such BCIs, and the number of subjects participating in such studies remains small and unrepresentative. 

Meanwhile, new approaches depart from the established matrix presentation, oftentimes with the triggered symbols placed at the center of the screen. Thus, users have the advantage that gaze shift is no longer needed, as shown in Acqualagna *et al.* (2010) [45] and Aloise *et al.* (2012) [46]. Although the T9 interface, suggested by Akram *et al.* (2014) [47], still is row and column based, it should be considered a new approach, as such an interface aggressively condenses the information and the size of the matrix, and with word suggestion this interface significantly outperformed the conventional ones. There is much potential in this direction of research, as it can better serve users with specific disabilities, and in terms of user acceptance. Comparison among different designs might be difficult to conduct, as these designs would serve different target groups. Thus, what matters is whether such designs can serve their purpose. We argue that user acceptance could serve as a universal measure of this. 

Finally, the hybrid approach is one of the more recent research directions. By adding more information collected simultaneously from participants, hybrid BCIs should be capable of processing at a higher bit-rate and performing more complex tasks. As shown in Ma *et al.* (2014) [50], users could control a multifunctional humanoid robot and a mobile robot. Researchers not only collected P300-based signals, but also captured other event-related potential components such as the N170 and the vertex positive potential (VPP), which were evoked by the configuration processing of facial images. Users’ eye movements, e.g., blink, wink, and gaze, were also recorded to assist the task. Riccio *et al.* (2015) [57] and Klobassa *et al.* (2009) [51] also explored the possibilities of combining EMG signals or auditory inputs with the conventional P300-based BCI. The former approach is especially promising for people with severe motor disability, while the latter could greatly benefit patients with visual difficulties. Hybrid approaches looked very promising in BCI applications, but with more components in the system, it will be a challenging research direction.

The following recommendations are made for future research:

1. After proof-of-concept studies, researchers need to conduct quantitative investigation on various configuration parameters, so that the prototype system can achieve better performance in terms of HFE. That is to say, having established proof-of-concept, while it is important to investigate further extensions or applications of the concept, possible improvements to its fundamental characteristics should not be ignored. Examination of approaches to yield incremental BCI improvement, and thus HFE improvement, can be incorporated into whole-system tests. For example, in a case such as Pathirage *et al.* (2013) [48], further investigation of the wheelchair-mounted robotic arm could incorporate testing of variations on the configuration of the non-uniform grid to see what configurations yield improved HFE, and thus overall system performance.
2. Comparison with the conventional paradigm is recommended as it would convey more information on performance metrics. In a manner similar to Aloise *et al.* (2012) [46] and Townsend *et al.* (2010) [44], comparing the performance of the well-established matrix oddball paradigm with variations or departures from it, as utilized by a common set of test subjects, allows the research community to develop baselines of BCI performance, and to better gauge the implications of new approaches to the conventional paradigm. For example, while the results of Akram *et al.* (2013) [47] are promising, they would be made stronger had the researchers conducted actual trials of the conventional speller with the test subjects, rather than only computing expected task times, in order to allow for proper comparison with their experimental results.
3. Although adequate sample size is not always possible, researchers should try to find more users that fit the specific usage of the BCI system. This is particularly important for designs that are targeting certain clinical groups. Of the eight articles reviewed for RQ1, only Townsend *et al.* [44] involved clinical participants, and then only three participants with ALS among 15 non-clinical undergraduates. That said, it cannot be assumed that all BCI research has in mind the development of BCIs for assistive use. If the research is simply of a general nature, then the selection of test subjects is not critical. However, if the research is intended to be of benefit to assistive technologies or to certain clinical groups, then it should be made explicit and every effort should be made to involve appropriate clinical participants in the research. If research has not been conducted with clinical participants, then it can only be considered as proof-of-concept, regardless of the degree of success or development of the underlying techniques or systems. Given that certain clinical groups have a small population in absolute terms (such as ALS patients), perhaps some studies could involve participants with more common disabilities to simulate rarer clinical groups. For example, if work extending on Aloise *et al.* (2012) [46] were to be directed explicitly at developing assistive technologies, for example for patients with a neurodegenerative disease, it could be conducted with participants with oculomotor impairments only. The use of such “proxy” participants might actually be preferable in some cases in order to reduce potential confounds, as some clinical groups present simultaneously a broad variety of capacities and challenges.

#### 5.1.2. Evidence for RQ2

Research Question 2 was “Within the constraints of BCI instrumentation, what environmental interventions (sedentary *vs.* ambulatory implementation, comfort of instrumentation, “normality” of acquisition method, *etc.*) can yield improved performance or satisfaction in subjects?” This question was concerned with findings that point toward system designs that could benefit HFE of BCI systems in terms of cognitive–affective response and adaptability to varying physical needs of users. Of the articles reviewed to address this question, four addressed system developments that could expand the applicability of P300-based BCI to real-world applications [34,51,53,57], one addressed the important issues of cost and simplicity—and thus availability and utility [54]—and three demonstrated potential real-world applications [52,55,56].

Castermans *et al.* (2011) [53] demonstrated that ambulatory systems are feasible, while finding that certain noise-correction methods did not add value. However, they were able to demonstrate the advantage of the xDawn spatial filter for future development. Riccio *et al.* (2015) [57] demonstrated a hybrid EEG–EMG system that reduced overall task time, and user workload and fatigue, thus indicating possibilities for development of systems better adapted to users with residual muscular activity. These studies speak to the issue of lessening cognitive load, which is crucial to good HFE for system performance. Both efforts concerned a hybrid approach to the BCI that used alternate pathways to reduce noise in the system and thus render signal acquisition more effective. In this way, the user’s gulf of execution is made smaller, thus permitting increases in performance and reduction in fatigue and stress. These two studies also expand possibilities with disabled users with mobility or residual motor activity. This is a very important element, as an important aspect of the HFE of a system is its adaptability to the needs of users. A similar HFE concern is adaptability to the user’s environmental context. Klobassa *et al.* (2009) [51] observed the challenges facing auditory systems, but found that continued development could yield benefits for users with impairments that prohibit the use of visual systems. By extension, this suggests possibilities for applications that would benefit from the relative omnidirectionality of sound, or where the user’s visual attention was otherwise engaged. Li *et al.* (2011) [34] demonstrated the importance of considering screen size in system design, demonstrating the importance of accounting for user capacities and preference in order to optimize system performance.

Duvinage *et al.* (2013) [54] recognized the need for low-cost and easy-to-use EEG equipment for the wide-spread application of P300-based BCIs. They identified the weaknesses of consumer-grade systems, but indicated that they do have a role for non-critical applications. They urged that low-cost, simpler systems be developed. However, it must be remembered that this is the perspective of researchers, not users. Simplicity, low encumbrance, and attractiveness should not be underestimated in terms of user acceptance. A less-than-perfect system that users are willing or eager to employ is better than a quality system that users resist. Cost should also be understood from an affective point of view, not simply a monetary one. Given the burdensome medical costs that disabled patients already face, and the emotional impact of such burdens, cost is not simply a monetary measure. The researchers should consider that perhaps accessibility has greater value to users than relative quality, given that assistive technologies must eventually exist “out in the world”, and not only in laboratories or well-equipped hospitals. 

Corralejo *et al.* (2014) [56] demonstrated a BCI system for the control of home devices. They had good results, and found that degree of impairment did not greatly impact the ability to make use of the system. Furthermore, they suggested that such a system was easy to adapt to the needs and requirements of users, and could reduce dependence on caregivers (which is of great benefit to users and caregivers alike). Tai *et al.* (2013) [55] demonstrated the integration of a BCI into a web browser, and suggested that, given further development, the ease of use of the system would allow for autonomous Internet browsing by impaired individuals. Finally, Escolano *et al.* (2010) [52] demonstrated a telepresence system through which an impaired individual could interact with the external environment through remote control of a robot that provided audiovisual feedback. They demonstrated that the system was advantageous in that, unlike other systems, it did not require sustained attention (which is difficult in real-world situations). Furthermore, the system had rehabilitative potential for impaired patients, and in addition the system was perceived as fun by users. The results of these studies suggest systems with good initial HFE, and point to a design process that considered the needs and capacities of the user. They also take an important step in the cognitive–affective direction by proposing systems that are more than assistive, but that also are empowering.

HFE are an implicit and important component in all of the studies reviewed for RQ2. The investigation of real-world applications, or techniques that will enable or improve real-world applications, necessarily entail crucial HFE. From this perspective, all of these studies are important contributions to understanding the HFE of P300-based BCI systems. These studies demonstrate the robustness of the oddball paradigm in adapting to a variety of user needs and context. Furthermore, they demonstrate that assistive applications can do more than simply improve system performance. Rather, they can empower users facing disabilities that themselves present terrible cognitive-affective burdens [41]. As such, the value of these developments is much greater than mere technical advancement. For able individuals, performance increases perhaps are easily measured quantitatively. But given that the application of the systems and methods presented in these studies are to the aid and benefit of disabled individuals, we must take greater account of the cognitive-affective aspects of user experience, as these aspects will have greater impact on performance than might be seen in use by able individuals. For this reason, we suggest that future research make explicit the connection with and assumptions of HFE in system performance in real-world or real-world-applicable contexts. Furthermore, researchers should take into account that HFE have greater importance for users that face challenges due to disease or misfortune that are not faced by the general population of users. While these are not strictly oversights, and are not a weakness in the current research, they are lost opportunities to extend the value and potential of these assistive technologies. This gap can be filled if researchers articulate clearly the link with HFE of their research, even if that research is not strictly concerned with HFE. They must capitalize on and leverage the importance of HFE considerations. 

The following recommendations are made for future research:

1. Make explicit the connections that real-world and real-world-applicable systems have to HFE. That is to say, BCI research is applied research, in that BCIs are tools that have human users, and which are intended to accomplish the users’ tasks. As such, HFE are an inescapable component of such research. HFE are always at play, regardless of whether or not researchers have addressed them. Thus, researchers must necessarily address them, as otherwise they become confounds. Addressing HFE explicitly will serve to reinforce such good practice in future work.
2. Acknowledge the value of attending to HFE of users who already face severe challenges in this regard. Similarly, understand that HFE might have greater importance for clinical groups than for non-clinical groups. For example, Li *et al.* (2011) [34] acknowledged this, and as well made an explicit connection with HFE, as they framed their work in the following manner:
  “For those with motor disabilities, a greater degree of communication and control can enhance their self-confidence and positive attitude toward life. For these reasons, BCI applications provide hope and encouragement for people suffering from neurodegenerative diseases …. The degree of communication and control afforded to people with motor disabilities by BCI helps not only to make numerous simple tasks more convenient but also to reduce the burden of their caretakers.”
3. Remember to view systems also from the perspective of users, and to evaluate them in this regard. Speaking directly to the question of cost *vs.* quality raised by Duvinage *et al.* (2013) [54], it could be that researchers should attend to the needs and capacities of users beyond what is evident in experimental settings. These needs and capacities furthermore often extend beyond individual users; Corralejo *et al.* (2014) [56] acknowledge this when they point out that increasing an individual’s autonomy and independence decreases dependence on nurses, caregivers and relatives. Researchers must keep in mind that unmeasured social or economic pressures might be as strong as any measured factors. Thus, HFE must be attended to, but they must also be expanded to incorporate socio-economic aspects of the user-system.
4. In making explicit the value of attending to HFE to improve system performance, be also sure to make explicit the value of design that avoids potential HFE deficits. For example, while Castermans *et al.* (2011) [53] were somewhat ambivalent about the value of their findings, stepwise improvements that avoid the potential pitfalls of sedentary or overly noisy systems are not neutral in HFE terms—they are a benefit. Similarly, Klobassa *et al.* (2009) [51] reported throughput rates that were not impressive compared to those possible with the visual paradigm, but they made the important point that visual mobility often is eventually impaired or even lost among certain clinical groups—thus, an otherwise excellent hypothetical system designed only according to the visual paradigm would offer nothing to such clinical groups.
5. Researchers must be aware that HFE for the disabled include more than the user—they also involve the user’s support network. Corralejo *et al.* (2014) [65] pointed out the benefit of their system not only to users, but also to caregivers. Similarly to point 3 above, researchers must have a social perspective on HFE for disabled users, as the human–machine system necessarily comprises more humans than the single user. Escolano *et al.* (2010) [52] acknowledged this in that they stated that their system was designed not only with the participation of patients (itself an essential factor), but as well with patients’ caregivers and family.
6. Researchers should attend more to the affective element of HFE. For example, Escolano *et al.* (2010) [52] were unusual in that they noted that their system was “fun”. This is not a word commonly seen in the literature. Nevertheless, researchers should not underestimate the potential value of positive affect to overall performance and acceptance. Indeed, it is important that a research agenda be targeted on the affective component of HFE, given the degree of influence of affect upon cognition (e.g., Forgas (2008) [68]). Corralejo *et al.* (2014) [56] and Tai *et al.* (2013) [55] could have given attention to the affective elements, given the potential of their systems for individual entertainment or human interaction. It is important that the research community makes a point to remember the importance of these more diffuse human needs, and not simply decompose those needs into component tasks. If we do that, then we risk decomposing users likewise into component tasks, and thus dehumanizing them (a risk which they often already face [41]).

#### 5.1.3. Evidence for RQ3

Research Question 3 was “Guided by research conducted in other assistive technologies, how can we measure user acceptance rates? Which concepts from HFE may increase user acceptance rates of P300-based BCI systems applied in assistive technology?” This question was concerned with identifying accurate metrics for user acceptance ratings and analyzing those metrics from the HFE perspective to make recommendations for further advancement of P300-based BCI assistive technology.

The viability of broad application of P300-based BCI technology depends on how the system can to be adapted to the needs of users, who can be categorized into three distinct groups based on their physical abilities: healthy users without disabilities, users with varying degrees of neuromuscular disabilities, and “locked-in” users. Additionally, according to the Technology Acceptance Model [69], each category will have different criteria for perceived usefulness and perceived ease of use of the new technology, as well as the subjective norm, which is related to mandatory or voluntary technology usage to perform tasks. We found that not only did most of the studies reviewed engage mostly or only with healthy participants, but some studies also did not disclose information about participants’ type of disability (e.g., [61]). Hence, one of our recommendations is that user acceptance evaluation of P300-based BCI interfaces should involve users of all three categories to produce valid and reliable suggestions for further design improvement. Out of five articles analyzed for this question, only two [59,62] directly addressed usability testing of P300-based BCI technology; the other three articles discussed usability and user acceptance of BCI technologies using different approaches: MI-based [61], SMR-based [60], and HF-SSVEP [58]. Our review found gaps in current research assessing P300-based BCI technology. Looking at the evaluation methods for the assistive BCI technologies described, we observed a heavier focus on operational as well as reliability- and maintainability-related objectives of HFE; system safety, accuracy, and performance were mentioned in every single design evaluation. However, user-related HFE objectives, such as increasing the ease of use and aesthetic appearance, were considered in very few studies. This could be due to the difficulties of identifying the appropriate metrics for such subjective but extremely important factors in user acceptance. Several studies approached usability assessment in accordance with ISO 9241-210 [67], measuring effectiveness (accuracy), efficiency (subjective workload and/or information transfer rate), and satisfaction (visual analogue scale, interview, Extended QUEST, and ATD-PA) [49,60,62]. Recognizing the value of such measurements, Riccio *et al.* (2011) [59] underlined the importance of applying HFE methods and objectives to assess BCI technology and recommended their implementation in further P300-based BCI research. We agree with these recommendations, and, in fact, we suggest that future research of all viable, reliable and high-performing assistive BCI systems include user acceptance testing applying the metrics discussed in this review.

Recognizing the importance of HFE objectives of focusing on the users, the BCI system operators, the studies reviewed for RQ3 envisioned usability assessment as a crucial step in the process of developing assistive technology that could adapt to the needs of the target user group; they engaged a limited number of subjects that represented the target audience, users with various neuromuscular disabilities. The focus on validity of the studies’ findings compromised their reliability, but even the degree of such external validity was limited. This results in limitations of their user acceptance findings, particularly, due to the complexity of experimental setup, as most studies were conducted in unnaturally simplified laboratory environments. Even Zickler *et al.* (2013) [62], who recreated the most real life-like conditions with background noise and minor distractions in participants’ homes or at the Information Center for Supported Communication, recognized that the small sample size (*n* = 4) and the lack of evaluation of long-term home use limited the study, yielding only descriptive conclusions, without any grounds for an in-depth analysis. This constitutes a serious limitation for wide application and generalization of the study findings on the user acceptance rates of the tested P300-based BCI systems. There seems to be a cycle in developing P300-based BCI systems for assistive technologies with high user acceptance rates: to further advance this technology, there needs to be a broad public support and interest, which is only possible with increased user acceptance rates; but, at the same time, to improve user acceptance by conducting comprehensive user testing in real life-like environment and long-term home use, the technology needs to develop further to support faster performance, simpler use, and more independent operation (reducing the necessary involvement of trained technicians or caregivers).

In the research of user acceptance rates of an MI-based video game, Bonnet *et al.* (2013) [61] encountered the challenge of identifying the balance between immersion and simplicity for the feedback design. They stated that the feedback system in a video game was crucial to evaluate the participant performance, associated with the player’s enjoyment and motivation levels, but that more complex feedback systems of commercial-quality immersive video games could lead to increased mental workload. In addition, even though their experiment, which compared subject performance in a single-user *vs.* multi-user (collaborative) BCI interaction in gameplay and then two approaches in a multi-user setting (collaborative and competitive), did not identify any significant difference in the mean classification performance within the three tested approaches, seven out of 20 participants reported being distracted by the excessive feedback in the multi-user BCI interaction.

Holz *et al.* (2013) [60] found that overall usability metrics of SMR-based BCI were lower than those of P300-based BCI (as reported in other studies): the effectiveness of three (out of four) users was low to medium; efficiency (mental workload) was moderate; and, while users rated their satisfaction relatively high regarding weight, safety, and learnability of the device, they were dissatisfied with its dimensions, adjustment, ease of use, and speed. The dissatisfaction with the EEG cap and excessive electrodes that restrict movement was one of the main findings in the study of Lightbody *et al.* (2010) [50], who tested a HF-SSVEP-based BCI interface with a lead user and two user groups (healthy and disabled subjects). Workshops and questionnaires conducted in their study offered more insight into technical user requirements, such as integration of access to multimedia and development of interoperable systems, and general recommendations to improve training techniques and usability of IGUIs. The two reviewed studies that evaluated usability of P300-based BCI systems concluded with similar concerns expressed by their test subjects [59,62]. 

The following recommendations are made for future research:

1. The researchers working on advancing P300-based BCI in assistive technologies need to engage participants with disabilities, who represent the majority of the target user group. This is consonant with recommendation 3 of Section 5.1.1. However, that previous recommendation specifies the involvement of users that match the intended usage profile of the system. This recommendation more specifically stresses the participation of clinical groups. This is of great importance in research that addresses the question of user acceptance. For example, Riccio *et al.* (2011) [59] explicitly framed their research in the context of improving usability for those with severe motor disability. Yet, despite a comprehensive assessment of usability, which is laudable, the data were collected only from non-clinical participants. While the study did validate the utility of the HFE methodologies used, they did so only for healthy participants. As such methodologies generally have been developed by and for use on healthy individuals, we should not assume that they are immediately valid for non-healthy individuals (*cf.* Anderson 2009 [70]). The only way that the research community will be able to validate similar methodologies for clinical groups is through their participation in studies using existing methodologies, and having those methodologies adapted specifically to them.
2. To yield valid user acceptance data characterizing technology that is intended to become a part of day-to-day life of users with disabilities, researchers need to conduct their tests not only in the lab, but in a real life-like environment, with multiple distracting factors and complex user-system interactions. That is to say, researchers must allow for environmental confounds, which make up the fabric of life outside of the laboratory. Zickler *et al.* (2013) [62] is an excellent example of this: EEG signals were not cleaned of artifacts such as eye blinks (as the researchers assumed real-world use in an environment full of artifacts), and “Daily life distractions, such as family members entering the room or telephone ringing were deliberately not avoided to guarantee for highest possible external validity.”
3. The P300-based technology developers should attend to HFE concepts for design improvements, as the reviewed literature suggested, for instance: improved appearance of EEG caps, more conventional and user-friendly interfaces, simplified software installation processes, clarified training systems for software use, elimination of the need for participation of a trained EEG technician, interoperable systems development, and integration with other systems and devices. Holz *et al.* (2013) [60] is an excellent example of how to incorporate attention to such a design approach, and likewise how to measure user response and acceptance. While the researchers collected useful system performance data in various trials, it was in using the Extended QUEST 2.0 [64] to assess participants that the researchers learned that some users felt not only physically uncomfortable, but felt that they “look[ed] strange” in the EEG cap, or that they found the cabling distracting and uncomfortable, or that they desired a different training schedule, or that the EEG looked too much like the “hospital”. Users and researchers have different goals and needs; to ensure acceptance by users (and thus to be able to provide to them utility), researchers must not forget always to attend to those goals and needs, and to ask what they are instead of making assumptions.

### 5.2. Review Limitations

This review was limited by the following factors:

- The literature search was conducted only in English.
- The generalizability of the findings is hampered by the specificity of the concern (*i.e.*, assistive technologies for individuals with certain neuromuscular impairments), and the resultant small number of representative test subjects (in our reviewed studies, n ranged only from 1 to 20).
- The bulk of research on P300-based BCI still addresses fundamentally technical issues, thus studies explicitly accounting for HFE are relatively few.
- Even among those studies addressing HFE, the goal is still technical (*i.e.*, performance-oriented), as much technical development remains to improve the functioning of P300-based BCI systems, so that focus can be given to refining HFE. Despite the fact that these issues are related, HFE remain a secondary concern.
- It is difficult to isolate evocation methods and environmental/design variables, as each category impacts the other.
- Attention to user acceptance is confounded by the fact that many target users are impaired in ways and in degrees that leave them little choice in assistive technologies. And even though most authors of the articles reviewed for RQ3 agree on the importance of applying HFE methods to assess BCI technology in terms of subjective workload (NASA TLX), user satisfaction measurement, and accuracy, user acceptance is unfortunately often neglected.

## 6. Conclusions

We reviewed a sample of the current literature on P300-based BCIs from an explicit HFE perspective. To evaluate what makes for good HFE in such systems, we focused on alternative implementations of the oddball paradigm (the machine focus), on environmental interventions (the human-machine focus), and on measurement of user acceptance (the human focus). In each focus, the review was grounded in HFE concepts, and in how system performance (*i.e.*, machine and human together) and satisfaction (not only of users, but also of the users’ social and care networks) can benefit from the inclusion of an explicit HFE perspective. 

We find that HFE concerns are central to the performance of these systems (more accurately *systems of systems*, given the social and technological complexity), although researchers do not often make explicit this connection. Furthermore, the variety of possible assistive implementations [71], as well as the variety and variability of user capacities and preferences [72], suggest that there is no universal metric or “best practice” to describe HFE in BCI applications, aside from urging the systematic inclusion of HFE. Each system must be tailored to users and contexts. As such, this argues for a general awareness of and attention to HFE in BCI system research. In future studies, this will necessitate an incorporation of measures of user acceptance, and rigorous usability evaluations (perhaps based on ISO 9241-210 [67]). As well, despite the relatively small percentage of the population with disabilities that would benefit from BCI assistive technologies, it is crucial that future research incorporate such potential users as test participants, as their HFE needs differ substantially from the able population. Along these lines, greater realism in testing is necessitated. That is, even techniques or systems that are in their infancy should be tested in situations that strive to mimic or replicate real-world contexts, as this will drastically impact the cognitive–affective state of users—a crucial concern for HFE. Finally, future research that explicitly addresses HFE must expand its understanding of this topic to incorporate socio-economic concerns, as well as user’s support networks.

The 12 recommendations put forward here provide a foundation from which could be developed a framework for the systematic and consistent inclusion of HFE concerns into research on P300-based BCIs, assistive technologies using BCIs, and assistive technologies in general. The relatively nascent state of many of these technologies should not prevent researchers from looking forward to possible and eventual real-world applications, as an HFE perspective will benefit not only outcomes, but as well the *processes* that lead to those outcomes. Additionally, speaking generally to the development of BCI, a systematic HFE perspective could help to anchor and to cohere the overall endeavor as it grows further beyond the laboratory [41,70,73,74].
